# Eco-Friendly Extraction of Olive Leaf Phenolics and Terpenes: A Comparative Performance Analysis Against Conventional Methods

**DOI:** 10.3390/foods14173030

**Published:** 2025-08-29

**Authors:** Lucía López-Salas, Xavier Expósito-Almellón, Anderson Valencia-Isaza, Alejandro Fernández-Arteaga, Rosa Quirantes-Piné, Isabel Borrás-Linares, Jesús Lozano-Sánchez

**Affiliations:** 1 Department of Food Science and Nutrition, University of Granada, Campus Universitario s/n, 18071 Granada, Spain; lucialpz@ugr.es (L.L.-S.); expositox@ugr.es (X.E.-A.); 2 Department of Chemical Engineering, Faculty of Sciences, University of Granada, Avda Fuentenueva s/n, 18071 Granada, Spain; avalenciai@unal.edu.co (A.V.-I.); jandro@ugr.es (A.F.-A.); 3 Department of Analytical Chemistry, Faculty of Sciences, University of Granada, Avda Fuentenueva s/n, 18071 Granada, Spain; iborras@ugr.es

**Keywords:** *Olea europaea* L., olive leaves, olive waste, polyphenols, terpenic compounds, HPLC-MS

## Abstract

The present study focuses on recovering phenolic compounds and terpenes from olive leaves, which are generated as by-products during olive oil processing. To this end, conventional extraction/maceration (CE) and advanced extraction techniques such as subcritical water extraction (SWE), pressurized fluid extraction (PLE) and ultrasound-assisted extraction (UAE) were employed to compare and determine the most effective procedure. The phenolic and terpenoid composition of the extracts revealed a total of 33 compounds in HPLC-QTOF-MS analysis. According to these findings, the optimal extraction techniques for the maximum recovery of secoiridoids from olive leaves were PLE and UAE, with no significant difference between them (21.9891 ± 2.5521 mg/g DW and 21.0888 ± 1.3494 mg/g DW, respectively). Regarding to flavonoids, UAE was the most effective extraction technique, yielding 4.9837 ± 0.6739 mg/g DW. However, SWE recovered the highest amount of phenolic alcohols (7.4201 ± 0.9848 mg/g DW), which could be due to degradation of the secoiridoids during extraction. Conversely, UAE was more successful than the other techniques for the extraction of the terpene family (0.7373 ± 0.0601 mg/g DW). The present study therefore focuses on comparing different extraction techniques for revalorizing olive leaves as a source of bioactive compounds, specifically polyphenols and terpenes, due to their beneficial health properties.

## 1. Introduction

Europe is the world’s leading producer of olive oil, with Spain, Italy, Portugal, Greece, and Turkey being among the main producers [1]. The importance of olive oil in the food industry is due to its multiple health benefits, particularly those associated with consuming extra virgin olive oil (EVOO). Consumption of this functional food has been related to the prevention of cardiovascular and inflammatory diseases, cognitive decline, and oxidative stress, among other actions [2,3]. These benefits are attributable to its bioactive composition, which possesses antioxidant, anti-inflammatory, and anticarcinogenic properties, among which phenolic compounds and terpenes stand out [4,5]. In this regard, the European Food Safety Authority (EFSA) has approved a health claim relating to olive oil polyphenols and their protection of blood lipids against oxidative damage [6]. This health claim refers to hydroxytyrosol and its derivatives, such as tyrosol and oleuropein. It is important to note that the regulation specifies that this approved health claim can only be used for virgin olive oils containing at least 5 mg of hydroxytyrosol and its derivatives per 20 g of olive oil (or 250 mg/kg) [6].

The presence of these phytochemicals, along with their bioactive properties, has also been reported in by-products derived from olive oil production [7,8,9]. Oleuropein is the most abundant polyphenol in the olive leaf [10,11]. This secoiridoid is notable for its antioxidant effects, as it protects cells from oxidative damage and reduces inflammation. This contributes to the prevention of chronic diseases, such as cancer, cardiovascular, neurodegenerative, and metabolic diseases [12,13]. Hydroxytyrosol has also proven to be an interesting health-related compound due to its antioxidant, anti-inflammatory, antitumor, and antimicrobial properties, among others [14,15]. Apart from oleuropein and hydroxytyrosol, other compounds of interest in olive leaves include ligstroside (a secoiridoid) and luteolin-glucoside (a flavonoid) [16,17,18].

Similarly, olive terpenes present in leaves and other by-products of the olive tree have also been shown to possess beneficial effects on human health. They have been noted for their antioxidant, anti-inflammatory, cardioprotective, antitumor, and neuroprotective activity [19,20]. For instance, these compounds may help to reduce cholesterol and blood pressure, and they are potential inhibitors of enzymes related to neurodegenerative diseases, reducing inflammation and oxidative damage [19,20].

As food by-products are still rich in valuable compounds, the revalorization of olive by-products, specifically leaves, is a key strategy for transforming olive industry waste into high-value ingredients, with applications in functional foods, cosmetics, and nutraceuticals. It is estimated that olive leaves account for around 10% of the total weight of the olives arriving at the mill [10]. The main sources of these leaves are pruning and harvesting of olive trees. Given the aforementioned composition and the quantity of this by-product generated during the production of olive oil and agricultural practices, research into the use of leaves as an economical source of natural bioactive compounds is rather promising.

In line with the principles of the circular economy and green chemistry, various advanced extraction techniques have been employed to recover these compounds from food by-products. Among these techniques, ultrasound-assisted extraction (UAE), pressurized fluid extraction (PLE), and subcritical water extraction (SWE) have advanced significantly in recent years. UAE is simple to implement, requires accessible equipment, and allows optimization of parameters such as time and potential. This makes it an ideal technique for laboratories, as it is easy to use and has low technical requirements [21,22]. It is also easily scalable since industrial ultrasound systems are available; however, it should be noted that extraction efficiency may decrease in large volumes if ultrasonic energy transmission is not optimized [22,23]. In general, UAE requires low operating and maintenance costs, although the initial investment depends on the scale of the equipment [21]. On the other hand, PLE requires more specialized equipment and greater control of parameters such as pressure and temperature, but offers high automation and reproducibility. It is a scalable and automatable extraction technique with well-established industrial applications, and it can process large volumes of raw materials continuously and efficiently [22,24]. However, its implementation requires a higher initial investment due to the need for pressurized and automated equipment [22,24]. Finally, SWE, like the previous technique, requires reactors capable of operating at high pressure and temperature, which implies a greater investment and the need for higher safety precautions. Although using water as a solvent would reduce cost, operating at higher temperatures and pressures than PLE poses technical and safety challenges, as well as requiring a significant initial investment in high-pressure reactors and safety systems [25,26].

In short, developing environmentally friendly extraction techniques is a responsible choice and a strategic necessity in a world that demands sustainable, safe, and efficient processes. These technologies enable the extraction of value without compromising the environment or human health, thereby aligning with the principles of green chemistry and the Sustainable Development Goals (SDGs). On the other hand, olive by-products, such as leaves, are rich sources of bioactive polyphenols and terpenes, opening up numerous opportunities for their revalorization into high-value-added products. This activity promotes the use of resources, reduces the environmental impact of agricultural waste, and creates new opportunities for innovation and sustainable development within the agri-food chain.

Therefore, this study aimed to compare the yield and recovery efficiency of olive leaf polyphenols and terpenes using green extraction techniques (PLE, SWE, and UAE) versus conventional methods. As far as we are concerned, this is the first study to carry out a comparison of the terpene content recovered with these technologies from olive leaves. It was expected that green extraction techniques would offer higher yields and more efficient recovery of terpenes and polyphenols from olive leaves compared to conventional extraction processes, due to their higher selectivity and ability to preserve bioactive compounds.

## 2. Materials and Methods

### 2.1. Samples

The *Olea europaea* leaves used in this study belonged to the Hojiblanca variety and were harvested in February 2023. The samples were supplied by Sociedad Cooperativa Andaluza Olivarera Pontanense, located in Puente Genil (Córdoba, Spain). Prior to extraction and analysis, the leaves underwent a traditional drying process under controlled conditions for 20 days, protected from light, to ensure optimal preservation. Subsequently, they were ground in a conventional mill until a fine and homogeneous powder was obtained, after which they were stored at room temperature in airtight, light-resistant containers.

### 2.2. Chemicals

For the extraction, ethanol was purchased from Fisher Scientific (Waltham, MA, USA), whereas double-deionized water was obtained with a Milli-Q system (Millipore, Bedford, MA, USA). Standards with purity higher than 98% (*w/w*) of trans-*p*-coumaric acid, hydroxytyrosol, tyrosol, and oleuropein were procured from Sigma-Aldrich (St. Louis, MO, USA); whereas luteolin-7-O-glucoside, loganin, and verbascoside were acquired from Extrasynthese (Lyon, France), and apigenin was purchased from LGC (Teddington, Middlesex, UK).

### 2.3. Extraction of Polyphenols, Terpenes, and Other Polar Compounds from Olive Leaves

The comparative analysis of the different extraction techniques to recover bioactive compounds was based on the previously optimized conditions when those were applied to the same olive leaf matrix. Conventional and advanced extraction of bioactive compounds was performed according to the conditions reported below. These conditions were already identified as optimal by the original researchers for each extraction technique. These previous studies evaluated the specific operational/equipment parameters as the most critical factors to the recovery of bioactive compounds for their respective techniques, thereby providing the optimal conditions to maximize the recovery of bioactive compounds. Among all the best extraction conditions, a specific sample-solvent ratio was described for each extraction technique and reproduced in this work in combination with the rest of the optimized parameters.

#### 2.3.1. Conventional Extraction (CE)

The CE was performed using the optimized experimental procedure established in a previous study, with slight modifications [7]. Target compounds were isolated using ethanol and water as solvents in two sequential steps. First, 1 g of the milled olive leaf sample was mixed with 10 mL of ethanol under continuous stirring at 300 rpm for 4 h (Innova 2300 Platform Shaker, New Brunswick Scientific, Eppendorf AG, Hamburg, Germany). After that, the mixture was centrifuged at 4000 rpm for 15 min (Rotofix 32 A, Andreas Hettich GmbH & Co.KG, Tuttlingen, Germany), after which the supernatant was collected. Subsequently, the solid residue was mixed with 10 mL of water, and the sample was soaked and centrifuged under the same conditions described above. Then, the ethanolic and aqueous supernatants were combined and filtered using 0.45 μm syringe filters.

#### 2.3.2. Subcritical Water Extraction (SWE)

For the SWE, the optimal protocol established by [27] was followed, with some modifications. The process was conducted in a Berghof BR-300 high-pressure batch reactor (Berghof Products + Instruments GmbH, Eningen unter Achalm, Baden-Württemberg, Germany), equipped with a Heidolph ZR-2000 stirring system (Heidolph Scientific Products GmbH, Schwabach, Germany) and a BHM heating jacket. All experiments started at an initial manometric pressure of 0 Pa. Temperature control was managed using a REX-C100 controller (RKC Instrument Inc., Tokyo, Japan) in PID mode. Thus, 17 g of olive leaf powder were extracted with 238 mL of distilled water at 220 °C for 30 min at a pressure of 2.4 MPa and continuous agitation. The extract was recovered by filtration with the aid of a vacuum pump and centrifuged under the conditions previously mentioned.

#### 2.3.3. Pressurized Liquid Extraction (PLE)

The phytochemicals were extracted from olive leaves following the optimized procedure established in research by [8], with minor variations. Experimental assays were performed using a Dionex ASE 100 Accelerated Solvent Extractor (Dionex Corporation, Sunnyvale, CA, USA). The 34 mL extraction cell was filled with a mixture of 4 g of olive leaf sample and inert sand (ratio 1:4, *w*/*w*) to increase the contact surface area, thereby improving extraction efficiency. This mixture was placed between two cellulose filters, with 3 g of sand at each end. The extraction was carried out at 105 °C for 5 min using ethanol as solvent, and at a pressure of 8.96 MPa, thanks to the use of nitrogen. The resulting extracts were cooled in an ice bath, then centrifuged under the aforementioned conditions and filtered using 0.45 μm filters.

#### 2.3.4. Ultrasound Assisted Extraction (UAE)

The equipment used for UAE was an Ultrasonic Processor UP400St (Hielscher Ultrasonics, Teltow, Germany) equipped with a 7 mm sonotrode. The experimental conditions were determined according to a previously reported method, with some modifications [28]. Briefly, the extracts were prepared by adding 50 mL of a hydroalcoholic mixture (70:30, EtOH-H_2_O, *v*/*v*) to the olive leaf sample (3 g) and immersing it in an ice bath. The extraction was carried out at an amplitude of 30% and with 25 J·mL^−1^ of energy applied to the sample. The total run time was 5 min, with a final temperature below 40 °C. The obtained extracts were then centrifuged and filtered under the conditions previously described.

All the extraction experiments (CE, SWE, PLE, and UAE) were performed in triplicate to guarantee process reproducibility. All the extracts obtained following the procedures mentioned above were brought to dryness in a rotatory evaporator at 39 °C (Waterbath R-124, Buchi, Barcelona, Spain). Then, dry residues were reconstituted in the specific extraction solvent. The extracts were stored in the absence of light and refrigerated at −20 °C until their analysis. Table 1 shows a comparison of the extraction conditions used in each technique.

### 2.4. Determination of Extraction Yield

The extraction yield of each extraction technique was calculated by considering the weight of the dried extract obtained and the amount of olive leaf used in the extraction process (Equation (1)):

(1) $$Yield\;\left(\%\right)=\frac{Weight\;of\;dried\;extract\;(g)}{Weight\;of\;dried\;leaves\;used\;(g)}\times 100$$

It should be considered that the calculated total extraction yield refers to the “crude extract” and not exclusively to phenols and terpenes, since the extract may contain a wide variety of other compounds, such as carbohydrates or fibers, in addition to these target analytes.

### 2.5. HPLC–QTOF Analysis

To identify and quantify the detected analytes in the olive leaf extracts (CE, SWE, PLE, and UAE), the samples were prepared at concentrations of 10 mg/L and 1 mg/L. The olive leaf samples were analyzed following a previously described method [29]. The instrument used for the analytical characterization was an Elute Plus LC Series UPLC coupled to a Compact QTOF mass spectrometer through an electrospray interface (ESI) from Bruker Daltonics (Bremen, Germany). Chromatographic separation was performed using a Zorbax Eclipse Plus C18 analytical column (150 mm × 4.6 mm i.d., 1.8 μm, Agilent Technologies, Palo Alto, CA, USA). The compounds were eluted using a previously reported multistep linear gradient of water with 0.25% acetic acid (eluent A) and methanol (eluent B) [29]. The flow rate was 0.5 mL/min, and the injection volume was 10 μL. The column temperature was kept constant at 25 °C throughout the analysis. The compounds eluting from the column were detected in negative ionization mode, with a mass detection range of 50–1300 *m*/*z*. The source operating conditions were: capillary voltage +4.5 kV, drying gas temperature 220 °C, drying gas flow rate 9 L/min, and drying gas pressure 3 bar. Prior to the injection of each sample, the mass analyser was externally calibrated using sodium formate clusters (5 mM sodium hydroxide dissolved in a 1:1 (*v*/*v*) mixture of water/propan-2-ol with 0.2% formic acid) in high precision calibration (HPC) mode [29].

For the data analysis, the acquired information was processed using Data Analysis 6.0 and TASQ Analysis software version 2023 (Bruker Daltonik, Bremen, Germany), whereas the acquisition was performed using HyStar 3.2 software (Bruker Daltonik). Identification was based on information provided by the instrument (retention time, exact mass, isotopic distribution, and molecular formula), and was compared with available standards, as well as with previously published research and databases. The quantitation of phenolic and terpenoid compounds was carried out using seven commercial standards: hydroxytyrosol, tyrosol, *p*-coumaric acid, oleuropein, verbascoside, luteolin-7-glucoside, and loganin. These standards enabled either direct quantitation (when the compound was present in the sample and matched the standard) or indirect quantitation (using structurally related calibration curves when no commercial standard was available). This approach is commonly applied in phytochemical analyses when full standard coverage is not feasible due to limited commercial availability. Standard stock solutions (1000 mg/L in methanol) were prepared individually and stored at −32 °C in the dark. Then, solutions at different concentrations within the 1–15 mg/L range of these standards were prepared in the same solvent. All the calibration curves showed good linearity (Table S1). The concentrations of phenolic compounds and terpenes in the samples were determined by measuring the peak area of each compound in the chromatogram. These values were then interpolated using the calibration curve equation of the corresponding commercial standard, if available; otherwise, the equation of a structurally similar compound was used. Thus, phenolic acids were quantified using the *p*-coumaric acid calibration curve. Regarding phenolic alcohols, hydroxytyrosol standard was used to quantify this compound as well as hydroxytyrosol glucoside, while tyrosol was used to quantify the latter compound. All secoiridoids were quantified using the oleuropein calibration curve, and verbascoside was used to quantify this compound. All glycosylated flavonoids were quantified using luteolin-7-glucoside, flavonoid aglycones with apigenin, and loganin was used for the quantification of all terpenes.

### 2.6. Statistical Analysis

The extraction of phenolic compounds and terpenes from olive leaves was conducted in triplicate using various methods. The resulting data were statistically analyzed using SPSS (version 28, IBM SPSS Statistics, Armonk, NY, USA) to detect the statistical differences in the extraction efficiency between the techniques. One-way ANOVA and Tukey’s test (95% confidence interval, *p* ≤ 0.05) were applied to assess significant differences in the amounts of key bioactive compound groups present in the olive leaf extracts. In addition to mean comparisons, effect sizes were calculated using η^2^ to estimate the proportion of variance explained by each extraction technique for the different bioactive compound families.

## 3. Results

### 3.1. Identification of Phenolic and Terpenic Compounds by HPLC–QTOF-MS

**Figure 1:**
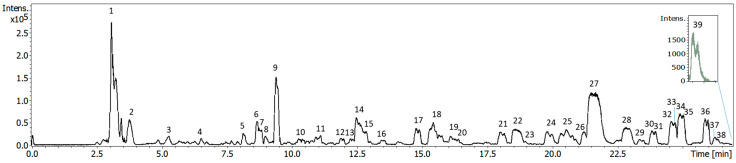
The BPC of UAE extract is representative of the analyzed samples obtained by HPLC-QTOF-MS.

Figure 1 illustrates a representative base peak chromatogram (BPC) obtained from the analyzed extracts in negative ionization mode. The compounds were identified using the chemical information from the HPLC-QTOF-MS analysis, supported by comparisons to available standards or reported data in the literature. In this sense, most of the compounds present in the different extracts were identified, a total of 33 compounds were characterized, whereas 3 molecules remained unknown.

Table 2 summarizes the tentative identification of the extracts, including the proposed compounds, their molecular formulae, their retention times (RT), their experimental and theoretical mass-to-charge ratios (*m*/*z*), the error (ppm), and the extraction techniques applied to each extract. All the tentatively identified compounds have previously been detected in olive samples [7,8,18,30,31,32,33,34,35,36].

With regard to the phenolic compounds, phytochemicals belonging to phenolic acids, alcohols, secoiridoids, and flavonoids were identified. A hydroxycinnamic acid, specifically *p*-coumaric acid (peak 20, *m*/*z* 163 and molecular formula C_9_H_8_O_3_), was characterized [18,31]. Concerning phenolic alcohols, tyrosol (peak 12, *m*/*z* 137 and molecular formula C_8_H_10_O_2_), hydroxytyrosol (peak 9, *m*/*z* 153 and molecular formula C_8_H_10_O_3_), and its derivatives hydroxytyrosol glucoside (peak 6, *m*/*z* 315) and two isomers of oxidized hydroxytyrosol (peak 3 and 13) were identified [7,8,30,31].

Pertaining to the secoiridoid group, 6 isomers of oleuropein (peaks 25, 27, 28, 31, 34, 35, with *m*/*z* 539 and molecular formula C_25_H_32_O_13_) were detected, together with its derivatives hydroxyoleuropein (peak 18, *m*/*z* 555) and oleuropein diglucoside (peak 26, *m*/*z* 701). In addition, 3 isomers of ligstroside (peaks 30, 32, 33, with *m*/*z* 523 and molecular formula C_25_H_32_O_12_) were also found. Other research studies about olive leaf have already identified numerous oleuropein and ligstroside isomers [32,33,34].

Moreover, regarding the flavonoid group, rutin (peak 24, *m*/*z* 609 and molecular formula C_27_H_30_O_16_), apigenin (peak 38, *m*/*z* 269 and molecular formula C_15_H_10_O_5_), luteolin (peak 37, *m*/*z* 285 and molecular formula C_15_H_10_O_6_), and 4 isomers of its derivative luteolin-glucoside (peaks 21, 22, 23, 29) were identified [18,31]. On the other hand, verbascoside (peak 19, *m*/*z* 623 and molecular formula C_29_H_36_O_15_), belonging to the phenylpropanoid/phenylethanol family, was also identified in the samples [30].

Apart from the aforementioned phenolic families, other compounds related to non-phenolic derivatives, such as oleoside and elenolic acid groups, were characterized. Indeed, 2 isomers of oleoside (peaks 7 and 14, with *m*/*z* 389 and molecular formula C_16_H_22_O_11_) and other compounds derived from elenolic acid, such as glucopyranosyl acyclodihydroelenolic acid (peak 10 and *m*/*z* 407) and elenolic acid glucoside (peak 15 and *m*/*z* 403), were also detected in these samples [7,8,30].

Regarding the terpenes group, 2 isomers of loganic acid (peaks 5 and 8, *m*/*z* 375 and molecular formula C_16_H_24_O_10_) were identified as in previous studies [33]. In addition, 7-epiloganin (peak 11, *m*/*z* 389 and molecular formula C_17_H_26_O_10_), lamiol (peak 17, *m*/*z* 377 and molecular formula C_16_H_26_O_10_), and maslinic acid (peak 39, *m*/*z* 471 and molecular formula C_30_H_48_O_4_) were also characterized [7,35].

Finally, other polar compounds classified within the group of sugars and derivatives were detected in these extracts: glucuronic acid (peak 1 and molecular formula C_6_H_12_O_7_) and sucrose (peak 2 and *m*/*z* 341) [36].

### 3.2. Quantification of Phenolic and Terpenic Compounds by HPLC–QTOF-MS

Table S1 provides details of the commercial standards applied, including their calibration ranges, calibration equations, R^2^ values, and LOD and LOQ for the analytical method. Table 3 showed the yield and the total bioactive compound content for the dried olive leaf obtained by CE, PLE, SWE and UAE, expressed as mg/g dry weight (DW). With regard to the extraction yield, these results pointed out that the different techniques obtained very similar yields, as there were hardly any significant differences between them. In this sense, SWE recovered a slightly higher quantity of extract while UAE presented the lower extraction efficiency. The extraction yields obtained in the present study were somewhat lower than the optimum achieved according to the literature, but they were within the expected range [8,37]. However, it should be taken into account that the extraction yield could not directly indicate the bioactive concentration of the extract. As mentioned above, the extraction yield refers to the total amount of material (dry extract) extracted from the starting sample (olive leaves) including non-target substances such as sugars, natural fibers, pigments, that could be extracted alongside the compounds of interest (phenolic compounds and terpenes). Therefore, a high total extract yield does not guarantee that the extract is enriched in specific target compounds, as shown in other studies of olive and other plant matrices [8,38].

The quantification of the phytochemicals revealed that PLE and UAE, both with lower extraction yields, succeeded in extracting the highest amounts of bioactive compounds, with no significant differences between them. It was expected that these extraction techniques would be able to recover high amounts of these bioactive compounds from the olive leaf, as previously demonstrated for several plant matrices [39,40,41,42,43,44]. It should be noted that SWE was the technique with the highest extraction yield (29 ± 5%), but with the lowest amount of bioactive compounds recovered (12 ± 1 mg compound/g DW olive leaf). This fact could be related to a degradation of the compounds of interest when extreme extraction conditions (220 °C and 30 min) were applied. Therefore, it was important to consider that very high temperatures for prolonged periods can affect thermolabile compounds such as polyphenols [40]. With regard to CE, it was already demonstrated in a previous study that it was an excellent option for the recovery of these phenolic and terpenic compounds [7], but it is important to consider other, more efficient, less polluting, and safer techniques.

**Figure 2:**
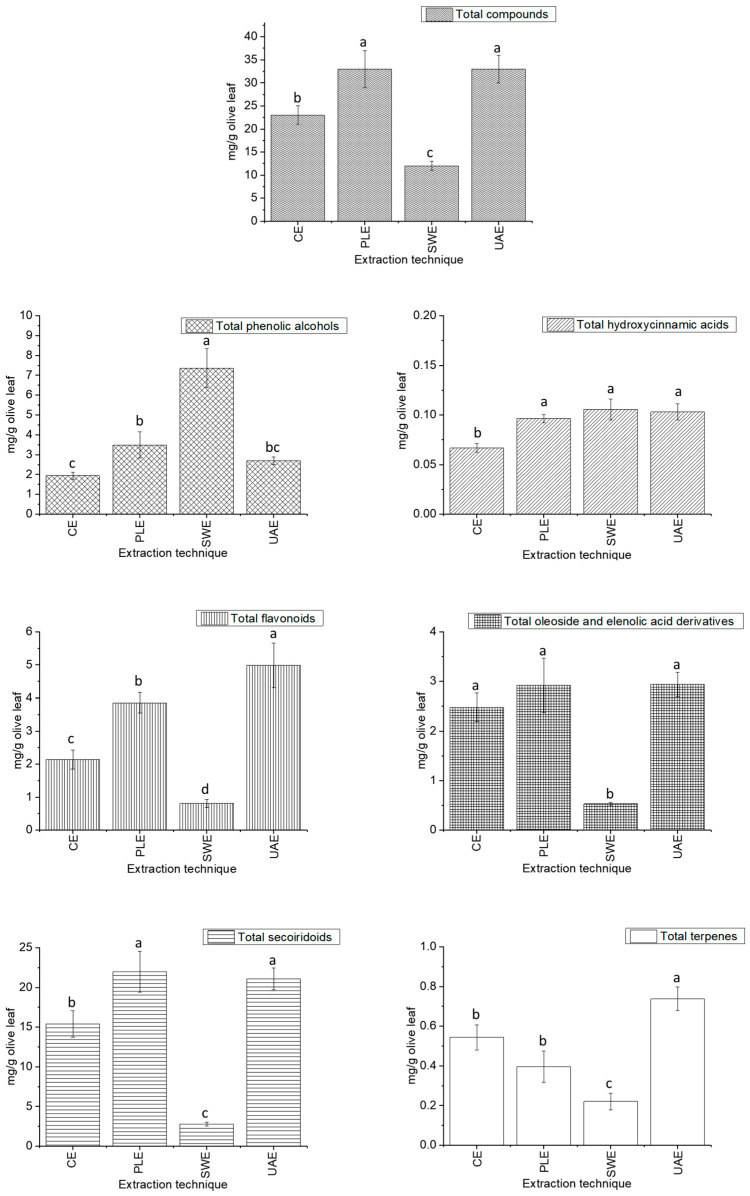
Quantification graphs of the total and individual compound families’ contents in olive leaf extracts. Error bars represent the standard deviation of the mean value (*n* = 3). Different letters in the same column indicate statistically significant differences (*p* ≤ 0.05) according to Tukey’s test.

Figure 2 depicted the total content determined for each of the families studied: phenolic alcohols, hydroxycinnamic acids, secoiridoids, flavonoids, oleosides, elenolic acid derivatives, and terpenes, under CE, PLE, SWE and UAE conditions. The concentrations were calculated as the sum of the quantified individual compounds by HPLC-QTOF-MS (Table S3) and expressed as the mean ± standard deviation, in mg of total compound per gram of DW.

These results suggest that the extraction technique has a significant effect on the concentration of bioactive compounds in olive leaf extracts, as supported by the analysis of the effect size (Table S2). Large effects (η^2^ > 0.87) were observed in all cases, being particularly high for flavonoids (η^2^ = 0.959), secoiridoids (η^2^ = 0.969), and phenolic alcohols (η^2^ = 0.949). These results demonstrate that the choice of extraction method significantly influences the composition of the extracts. Indeed, the recovery capacity of the different families of phenolic compounds and terpenes varied considerably among the four studied extraction techniques. SWE was the technique that showed the highest phenolic alcohol content, while the amounts of hydroxycinnamic acids recovered by SWE, UAE, and PLE did not differ significantly. The same occurred with the recovery of oleoside and elenolic acid derivatives, for which PLE, UAE, and CE obtained very similar quantities with no significant differences. Regarding secoiridoids, PLE and UAE obtained high and similar recovery rates. Finally, UAE emerged as the most effective procedure, demonstrating significant differences compared to the other extraction techniques in the recovery of flavonoids and terpenes from olive leaves.

Regarding hydroxycinnamic acids, the recovery of *p*-coumaric acid was very similar for the three green extraction techniques with no significant differences between them (SWE recovered 0.1054 ± 0.0105 mg compounds/g DW, UAE recovered 0.1030 ± 0.0081 mg compounds/g DW and PLE recovered 0.0964 ± 0.0041 mg compounds/g DW). On the opposite, CE was notable for its significantly lower content of this compound (0.0666 ± 0.0043 mg compounds/g DW). This is consistent with a previous study in which *p*-coumaric acid could not be quantified in olive leaf CE extracts [7].

Following with the secoiridoid group, the numerous oleuropein isomers stood out due to their high quantity relative to the other compounds of this family. Several studies indicate that oleuropein is the main secoiridoid present in olive leaves, making it the most interesting compound in this plant tissue [45]. The 3 isomers of oleuropein that eluted first were the most abundant in PLE, UAE and CE. However, they were not detected in SWE, possibly due to the higher concentration of phenolic alcohols found in this extraction technique compared to the others, as described below. Similarly, 3 isomers of ligstroside were detected and quantified in PLE, UAE and CE, but also undetected in SWE. Furthermore, although the PLE extraction had the highest secoiridoid content (21.9891 ± 2.5521 mg compounds/g DW), there was no significant difference compared to UAE (21.0888 ± 1.3494 mg compounds/g DW). These results demonstrate the effectiveness of both extraction techniques in recovering one of the most important polyphenol families from olive leaves.

In regards to the total phenolic alcohols, SWE extracted 7.4201 ± 0.9848 mg compounds/g DW, which is a much higher quantity than that recovered by other extraction techniques. This large amount of phenolic alcohols is directly related to the secoiridoid family. During the extraction process, if the conditions are too extreme for the thermolabile compounds, some of the secoiridoids can be hydrolysed. Specifically, oleuropein can be converted to hydroxytyrosol via enzymatic and/or chemical hydrolysis processes, particularly when high temperatures are employed to accelerate and promote this conversion [46,47]. The degradation of oleuropein at high temperatures has been studied in various contexts, revealing that this phenolic compound is susceptible to heat and can decompose, particularly in aqueous extracts and during heat treatments [48,49]. Studies of the degradation kinetics have pointed out that oleuropein in aqueous extracts follows first-order kinetics, resulting in a significant increase in the degradation rate over time and with temperature. In addition, low pH and/or the presence of enzymes such as β-glucosidase can accelerate the degradation of phenolic glycosides, even at lower temperatures [48,49]. In this sense, the combined effect of temperature, time and the presence of β-glucosidase enzymes on phenolic glycosides could be related to the composition of phenolic alcohols and secoiridoids in SWE extracts. In fact, these factors give rise to simpler phenolic alcohols, mainly hydroxytyrosol and tyrosol from oleuropein and ligstroside, respectively. The SWE extract showed the highest phenolic alcohol content, while the concentration of secoiridoids was extremely low compared to the other extraction techniques (2.7730 ± 0.2051 mg compounds/g DW). This makes even more sense, considering that the six oleuropein isomers, especially the three that are particularly abundant, were identified and quantified in all extraction techniques except SWE. Consequently, the amount of hydroxytyrosol quantified in SWE was much higher than in the other techniques (specifically double that in PLE and three times higher that in UAE). Additionally, the decrease in hydroxytyrosol glycoside concentration in the SWE extraction was noteworthy, as this compound was abundant in the other techniques. This decrease could indicate the hydrolysis of the sugar moiety. However, the possible oxidation of hydroxytyrosol to its oxidized form due to the extreme conditions of SWE should also be taken considered. Although the amount of the oxidized hydroxytyrosol isomer 2 (0.1785 ± 0.0359 mg compounds/g DW) was 20 times higher than that quantified in PLE (0.0166 ± 0.0006 mg compounds/g DW) or UAE (0.0090 ± 0.0018 mg compounds/g DW), it did not represent a significant proportion of the total hydroxytyrosol quantified in those techniques (2.0630 ± 0.4362 mg compounds/g DW in PLE and 1.3406 ± 0.0917 mg compounds/g DW in UAE). Therefore, there was no significant degradation of hydroxytyrosol to its oxidized form using the studied extraction techniques.

Furthermore, considering the PLE and UAE results, the hydroxythorosol and tyrosol contents were slightly higher in PLE, though there was no statistically significant difference (2.0630 ± 0.4362 and 0.1328 ± 0.0242 mg compounds/g DW, respectively). These amounts were in agreement with, and even higher than, those reported in the literature [8,50]. As expected, CE recovered the lowest amount of phenolic alcohols (1.9394 ± 0.1676 mg compounds/g DW), although there was also no significant difference compared to UAE (2.6894 ± 0.1854 mg compounds/g DW).

For the flavonoid family, significant differences were observed in the results of all extraction techniques. UAE recovered the highest amount of flavonoids (4.9837 ± 0.6739 mg compounds/g DW), followed by PLE (3.8554 ± 0.3109 mg compounds/g DW) and CE (2.4441 ± 2.1344 mg compounds/g DW These contents were similar to, or even higher than, those obtained in other studies [7,8]. As with other families, SWE was the least effective technique, recovering only 0.8073 ± 0.1244 mg compounds/g DW. Although luteolin-glucoside isomers were the most abundant of this family in UAE, PLE and CE, with luteolin-glucoside isomer 4 standing out, only three of these isomers were quantified in SWE in very small amounts. As with oleuropein, high extraction temperatures together with the possible activity of glucosidase enzyme, could explain the degradation of the flavonoid glucosides present in the olive leaf. Moreover, the low concentration of luteolin aglycone found in SWE suggests that the glycosylated forms were not degraded giving rise to the aglycone derivative. While thermal decomposition of flavonoid glycosides generally produces aglycones, some studies have documented additional degradation pathways involving the formation of simpler compounds in which the aglycone is not the main product. For instance, polyhydroxylated flavonoids and their glycosides can degraded via central ring opening when heated with water, generating simple aromatic compounds such as 1,3,5-benzenetriol, trihydroxybenzoic acids and trihydroxybenzaldehydes [51]. Therefore, in these cases, the aglycone form would not be the main product of the degradation, smaller molecules would be formed by flavonoid skeleton degradation instead. In this sense, previous research has confirmed that luteolin-glucoside is the main flavonoid recovered from olive leaves [52], which is consistent with the results obtained using other extraction techniques.

Regarding verbascoside, UAE (0.3625 ± 0.0285 mg compounds/g DW) achieved the highest recovery, followed by PLE (0.2077 ± 0.0382 mg compounds/g DW) and CE (0.1358 ± 0.0153 mg compounds/g DW). On the opposite, this compound was not detected in SWE. Following the same trend, analysis of the oleoside and elenolic acid derivatives group indicated that the recovery of these compounds was similar, with no significant difference between PLE (2.9202 ± 0.5507 mg compounds/g DW), UAE (2.9379 ± 0.2441 mg compounds/g DW) and CE (2.4825 ± 0.2858 mg compounds/g DW). Two oleoside isomers were the major compounds of this group for all the extraction techniques. Once again, SWE was shown to be the technique with the least recovery for these compounds, with 0.5311 ± 0.0291 mg compounds/g DW.

Finally, UAE showed the highest recovery of terpenes (0.7373 ± 0.0601 mg compounds/g DW), with lamiol being the major compound of this family (0.3825 ± 0.0306 mg compounds/g DW). This compound has previously been described as an interesting terpene in olive leaf [7,36]. Following UAE, there were no significant differences in terpenes content between PLE (0.3956 ± 0.0784 mg compounds/g DW) and CE (0.5438 ± 0.0636 mg compounds/g DW). Finally, SWE was the extraction technique with the lowest terpene recovery (0.2202 ± 0.0428 mg compounds/g DW), and the only one unable to extract maslinic acid.

## 4. Discussion

The data gathered showed that different conclusions could be drawn depending on the objective to be achieved, once the four different extraction methods had been studied. Taking into account the results obtained for UAE and PLE extractions, both techniques would be a good choice as there were no significant differences in the extraction of the compounds of interest in terms of total contents, as shown in Figure 2. However, a small, albeit insignificant, difference between these two techniques was observed in the extraction of phenolic alcohols and secoiridoids families, which were extracted in slightly higher quantities by PLE (Table S3). This similar behaviour is not surprising, given that phenolic alcohols and secoiridoids share part of their structure, with the former being derived from the latter. Therefore, to obtain the highest possible recovery of secoiridoids, PLE would be the green technique of choice. This conclusion is consistent with previous research, which concluded that this extraction technique was more effective in recovering this kind of polyphenol [53]. Secoiridoids are especially relevant for the nutraceutical and pharmaceutical industries due to their well-documented biological activities. Thus, they may contribute to formulations such as capsules, liquid extracts, or standardized powders aimed at preventing chronic diseases such as metabolic syndrome, cardiovascular disorders, and neurodegenerative conditions [12,13].

By contrast, SWE is undoubtedly the preferred extraction technique when it comes to phenolic alcohols. This is because these compounds are primarily the result of the breakdown of other glycosylated polyphenols, as discussed in the previous section. The results of the present study are consistent with those of previous studies, which have shown that polyphenols from olive and other plant sources, when extracted using the SWE technique, can degrade and undergo hydrolysis [27,54]. In this vein, recent research has focused on obtaining higher amounts of hydroxytyrosol by hydrolysing oleuropein in olive leaves [55,56]. Despite the good results obtained in these studies, the solvents used, including acids and organic solvents such as ethyl acetate, have not been qualified as GRAS. This highlights the importance of the present results for SWE. Therefore, SWE has proven to be an efficient, environmentally friendly, and GRAS-qualified option for producing target compounds resulting from the hydrolysis of plant polyphenolic glycosides, such as hydroxytyrosol from hydroxytyrosol glucoside and oleuropein, or tyrosol from ligstroside. Due to their high bioavailability and regulatory acceptance for health claims (EFSA) [6], phenolic alcohols are of particular interest in the nutraceutical and pharmaceutical industries. Moreover, their remarkable oxidative stability and solubility make them suitable for cosmetic formulations aimed at protecting the skin and combating ageing [57]. Furthermore, incorporating them into functional foods and exploring their potential role as natural preservatives in the food industry further extends their relevance.

When it comes to flavonoids, another important family of phenolic compounds, UAE is the technique of choice due to its high recovery rate and significant improvement in recovery. Another effective extraction technique for these compounds is PLE, although the difference with UAE is substantial. Since UAE is one of the main techniques used for flavonoid extraction, research has focused on optimising the extraction of this family of polyphenols from olive leaves individually [52]. Flavonoids are particularly important for the nutraceutical and cosmetic industries, where they help to prevent oxidative stress-related disorders and contribute to the development of anti-ageing and skin-soothing products [58]. In the food industry, flavonoids can serve as natural antioxidants in functional beverages and fortified products. Their relatively mild sensory impact, combined with high bioactivity, makes them suitable for direct inclusion in consumable formulations [59]. Finally, according to the results obtained, the SWE technique is not recommended for extracting flavonoids from olive leaves, since the recovery rate was minimal compared to other techniques. Furthermore, rutin was the only flavonoid extracted by SWE, whereas other techniques succeeded in recovering additional flavonoids, including luteolin-glucoside isomers.

However, in addition to polyphenols, the terpenes group was another objective of this study, giving novelty to this research. In this case, UAE provided the best results for these substances, showing significant differences with respect to the other techniques. Terpenes are highly valued for their aromatic properties and bioactivity. These compounds are particularly important in the cosmetics and fragrance industries, where they contribute to the sensory profile of formulation and offer antimicrobial, anti-inflammatory and soothing properties [60]. In the nutraceutical field, terpenes may act synergistically with phenolic compounds to enhance anti-inflammatory and antioxidant activity. Moreover, there is growing interest in their use as natural flavor enhancers or preservatives in functional foods and beverages [61]. CE was the second-best technique, achieving a significantly higher yield of terpenes than PLE and SWE. A previous study by this research group has already demonstrated the great capacity of CE to recover terpenes from the olive industry by-products [7]. Nevertheless, CE clearly has the disadvantage of being a polluting and non-harmless technique. For these reasons, UAE is evidently the preferred technique for recovering terpenes.

Taking into account all these results, UAE is considered the best extraction technique according to the objectives of this study. This choice is based on its selective efficiency and higher recovery of bioactive compounds, compared to the other evaluated technologies. The experimental results showed that UAE achieved the highest recovery of terpenes and flavonoids. This is highly relevant given the importance of these compounds in terms of their antioxidant, anti-inflammatory, and functional properties in natural matrices, such as olive leaf. In addition, while PLE exhibited high concentrations of phenolic alcohols and secoiridoids, UAE did not demonstrate significant variations in the quantity of these compounds compared to PLE. This confirms that UAE achieves selectivity and efficiency for key compound classes without compromising the recovery of others. Given its extraction yield, low operating temperature, and compatibility with green chemistry principles, UAE emerges as the most promising technique for applications in the nutraceutical, functional food, and olive leaf-based natural products industries, where the preservation and concentration of sensitive bioactives is essential.

## 5. Conclusions

The olive oil industry generates large quantities of by-products, including olive leaves. These leaves are a potential source of bioactive compounds with numerous health-promoting properties, as evidenced by the literature. In this regard, phenolic compounds and terpenes, in particular, have demonstrated high antioxidant and protective capacities against numerous diseases in other studies.

The present study aimed to compare different green and GRAS extraction techniques with conventional extraction, focusing on the recovery of various families of phenolic compounds and terpenes. These compounds were characterized qualitatively and quantitatively by HPLC-QTOF-MS.

It is important to note that, depending on the objective and the family of compounds to be extracted, the technique of choice may vary. In this case, SWE was the best technique for producing (from the secoiridoids already present) and extracting phenolic alcohols from olive leaf, with significantly higher recovery rates than the other techniques studied. However, the rest of the studied families could not be effectively recovered, possibly due to the extreme extraction conditions used. Due to the elevated temperature and dielectric properties of water in subcritical conditions, SWE can act as a chemical modification process, promoting the deglycosylation, hydrolysis, and structural transformation of complex phenolics. In addition, the presence of endogenous enzymes in the extract may contribute to the observed compound profile. Therefore, this extraction technique could be promising for clean-label food applications, where the exclusive use of water is advantageous and the transformation of complex precursors (e.g., into hydroxytyrosol) could be beneficial. For this reason, and particularly given its ability to produce hydroxytyrosol, this technique could be of interest to the nutraceutical, pharmacological, and cosmetic fields.

In general, PLE did not stand out for the recovery of any particular family of compounds, but it showed the highest contents of secoiridoids and elenolic acid derivatives. In fact, there were no significant differences in the recovery obtained by UAE for both families of compounds. However, this extraction technique could be particularly well-suited to high-value pharmaceutical and nutraceutical applications where higher concentrations of secoiridoids are desired, despite the higher energy requirements. Conversely, UAE was the most effective extraction technique for flavonoids and terpenes, the latter of which were a key focus of the present research. Although there were no significant differences in the total content of bioactive compounds in PLE and UAE, the ability of the latter to recover the highest amount of terpenes from olive leaves made the difference. Furthermore, UAE is ideal for nutraceuticals, cosmetics, and functional foods due to its mild conditions, high bioactive compound recovery, and preservation of the integrity of these compounds.

Therefore, UAE is the best choice for extracting bioactive compounds from olive leaves due to its selectivity, high recovery efficiency of target compounds (such as phenolic alcohols, secoiridoids, flavonoids, and terpenes), and favourable energy consumption, solvent use, and environmental impact. UAE operates at relatively low temperatures and pressures, uses small amounts of solvents, and allows the use of solvents qualified as green, while also requiring short extraction times. These properties make UAE highly compatible with green extraction principles and energy-efficient protocols.

## Figures and Tables

**Table 1:** Parameters of conventional and advanced extraction techniques.

Extraction Technique ^1^	Extraction Time (min)	Extraction Temperature	Extraction Pressure
CE	480	Room temperature	Atmospheric pressure
SWE	30	220 °C	2.4 MPa
PLE	5	105 °C	8.96 MPa
UAE	5	<40 °C	Atmospheric pressure

^1^ CE: Conventional Extraction; PLE: Pressurized Liquid Extraction; SWE: Subcritical Water Extraction; UAE: Ultrasound-Assisted Extraction.

**Table 2:** Tentatively identified compounds in the analyzed olive leaf extracts, numbered according to their retention time.

							Extraction Technique ^2^			
Peak	Proposed Compound	Molecular Formula	RT ^1^ (min)	*m*/*z* (exp)	*m*/*z* (theor)	Error (ppm)	CE	PLE	SWE	UAE *
1	Glucuronic acid	C_6_H_12_O_7_	3.09	195.0511	195.0510	−0.3	✓	✓	✓	✓
2	Sucrose	C_12_H_22_O_11_	3.78	341.1077	341.1089	3.7	✓	✓	✓	✓
3	Oxidized hydroxytyrosol isomer 1	C_8_H_8_O_3_	5.28	151.0398	151.0401	1.7	✓	✓	✓	✓
4	UK ^3^ 1	C_9_H_13_O_6_	6.52	217.0715	217.0718	1.2	✓	✓	✓	✓
5	Loganic acid isomer 1	C_16_H_24_O_10_	8.15	375.1311	375.1297	−3.7	✓	✓	✓	✓
6	Hydroxytyrosol glucoside	C_14_H_20_O_8_	8.68	315.1087	315.1085	−0.6	✓	✓	✓	✓
7	Oleoside/secologanoside isomer 1	C_16_H_22_O_11_	8.86	389.1095	389.1089	−1.4	✓	✓	✓	✓
8	Loganic acid isomer 2	C_16_H_24_O_10_	9.00	375.1286	375.1297	10.5	✓	✓	✓	✓
9	Hydroxytyrosol	C_8_H_10_O_3_	9.40	153.0553	153.0557	3.1	✓	✓	✓	✓
10	Glucopyranosyl acyclodihydroelenolic acid	C_17_H_28_O_11_	10.26	407.1575	407.1559	−3.9	✓	✓	✓	✓
11	7-Epiloganin	C_17_H_26_O_10_	11.10	389.1468	389.1453	−3.8	✓	✓	✓	✓
12	Tyrosol	C_8_H_10_O_2_	12.00	137.0617	137.0608	−6.2	✓	✓	✓	✓
13	Oxidized hydroxytyrosol isomer 2	C_8_H_8_O_3_	12.39	151.0402	151.0401	−0.8	✓	✓	✓	✓
14	Oleoside/secologanoside isomer 2	C_16_H_22_O_11_	12.43	389.1079	389.1089	2.5	✓	✓	✓	✓
15	Oleoside methyl ester/secologanoside methyl ester/elenolic acid glucoside	C_17_H_24_O_11_	12.53	403.1238	403.1246	2.0	✓	✓	✓	✓
16	UK ^3^ 2	C_21_H_33_O_10_	13.44	445.2068	445.2079	2.4	✓	✓	ND	✓
17	Lamiol	C_16_H_26_O_10_	14.75	377.1436	377.1453	4.6	✓	✓	✓	✓
18	Hydroxyoleuropein	C_25_H_32_O_14_	15.42	555.1699	555.1719	4.0	✓	✓	✓	✓
19	Verbascoside	C_29_H_36_O_15_	16.15	623.1844	623.1971	−3.0	✓	✓	✓	✓
20	Coumaric acid	C_9_H_8_O_3_	16.42	163.0382	163.0401	11.0	✓	✓	✓	✓
21	Luteolin-7-glucoside isomer 1	C_21_H_20_O_11_	18.03	447.0915	447.0933	7.0	✓	✓	✓	✓
22	Luteolin glucoside isomer 2	C_21_H_20_O_11_	18.58	447.0924	447.0933	1.0	✓	✓	✓	✓
23	Luteolin glucoside isomer 3	C_21_H_20_O_11_	18.85	447.0927	447.0933	1.1	✓	✓	ND	✓
24	Rutin	C_27_H_30_O_16_	19.79	609.1421	609.1461	6.6	✓	✓	✓	✓
25	Oleuropein isomer 1	C_25_H_32_O_13_	20.52	539.1737	539.1770	6.1	✓	✓	ND	✓
26	Oleuropein diglucoside	C_31_H_42_O_18_	21.19	701.2289	701.2298	12.3	✓	✓	✓	✓
27	Oleuropein isomer 2	C_25_H_32_O_13_	21.48	539.1735	539.1770	6.5	✓	✓	✓	✓
28	Oleuropein isomer 3	C_25_H_32_O_13_	22.80	539.1742	539.1770	5.2	✓	✓	ND	✓
29	Luteolin glucoside isomer 4	C_21_H_20_O_11_	23.32	447.0924	447.0933	2.0	✓	✓	✓	✓
30	Ligstroside isomer 1	C_25_H_32_O_12_	23.81	523.1791	523.1821	5.7	✓	✓	ND	✓
31	Oleuropein isomer 4	C_25_H_32_O_13_	23.94	539.1737	539.1770	6.2	✓	✓	✓	✓
32	Ligstroside isomer 2	C_25_H_32_O_12_	24.54	523.1806	523.1821	2.8	✓	✓	✓	✓
33	Ligstroside isomer 3	C_25_H_32_O_12_	24.70	523.1802	523.1821	3.6	✓	✓	ND	✓
34	Oleuropein isomer 5	C_25_H_32_O_13_	24.89	539.1745	539.1770	4.6	✓	✓	✓	✓
35	Oleuropein isomer 6	C_25_H_32_O_13_	25.01	539.1725	539.1770	8.3	✓	✓	✓	✓
36	UK ^3^ 3	C_27_H_38_O_15_	25.97	601.2106	601.2138	5.3	✓	✓	✓	✓
37	Luteolin	C_15_H_10_O_6_	26.35	285.0372	285.0405	11.6	✓	✓	✓	✓
38	Apigenin	C_15_H_10_O_5_	26.43	269.0425	269.0455	11.3	✓	✓	ND	✓
39	Maslinic acid	C_30_H_48_O_4_	27.16	471.3470	471.3480	2.2	✓	✓	ND	✓

^1^ Retention time, expressed in minutes. ^2^ CE: Conventional Extraction; PLE: Pressurized Liquid Extraction; SWE: Subcritical Water Extraction; UAE: Ultrasound-Assisted Extraction; ND: Non-detected. ^3^ UK, unknown compound. * All the values shown in the table refer to an extract obtained by UAE.

**Table 3:** Extraction yield and quantitative results of CE, PLE, SWE, and UAE extractions expressed in mg compound/g DW olive leaf as the mean ± standard deviation value (X ± SD).

Extraction Technique	Yield (%)	Total Bioactive Compound ^1^ (mg Compound/g Olive Leaf DW ^2^)
CE	26 ± 1 ^a^	23 ± 3 ^b^
PLE	19 ± 2 ^b^	33 ± 4 ^a^
SWE	29 ± 5 ^a^	12 ± 1 ^c^
UAE	24 ± 1 ^ab^	33 ± 3 ^a^

Different letters in the same column indicate statistically significant differences (*p* ≤ 0.05) according to Tukey’s test. ^1^ Total bioactive compound refers to the sum of all identified and quantified phenolic and terpenic compounds in each olive dried leaf extract. ^2^ DW: dry weight of olive leaves.

## Data Availability

The original contributions presented in this study are included in the article/Supplementary Materials. Further inquiries can be directed to the corresponding authors.

## Supplementary Materials

The following supporting information can be downloaded at: https://www.mdpi.com/article/10.3390/foods14173030/s1, Table S1: Standards and calibration curves used in the quantification of phenolic and terpenic compounds; Table S2: Effect sizes (η^2^) of the type of extraction technique on the content of bioactive compounds in olive leaf; Table S3: Quantification of compounds identified in olive leaf extracts.
