# Supplementary material for: Eco-Friendly Extraction of Olive Leaf Phenolics and Terpenes: A Comparative Performance Analysis Against Conventional Methods
Source: Foods. 2025 Aug 29;14(17):3030. doi: 10.3390/foods14173030 (PMC12428601; doi:10.3390/foods14173030)
Supplement: Supplementary file 1 [file foods-14-03030-s001.zip › foods-3750529-supplementary.pdf]

Table S1. Standards and calibration curves used in the quantification of phenolic and terpenic compounds.

Standard	Calibration Range (mg/L)	Calibration Curve	R^2	LOD[1] (mg/L)	LOQ[2] (mg/L)
		**Phenolic alcohols**			
**Hydroxytyrosol**	1 – 15	y = 41639 x + 10417	0.99	0.0267 ± 0.0001	0.0891 ± 0.0002
**Tyrosol**	1 – 15	y = 21121 x + 3767.5	0.98	0.1305 ± 0.0682	0.4351 ± 0.0227
		**Hydroxycinnamic acid**			
***p*-Coumaric acid**	1.6 – 15	y = 10819 x - 1534.9	0.98	0.4813 ± 0.0044	1.6043 ± 0.0148
		**Secoiridoid**			
**Oleuropein**	1 – 15	y = 38509 x + 37875	0.97	0.1692 ± 0.0045	0.5641 ± 0.0149
		**Phenylethanoid glycoside**			
**Verbascoside**	2 – 15	y = 13115 x - 4733.1	0.99	0.6214 ± 0.0273	2.0718 ± 0.0909
		**Flavonoids**			
**Luteolin-7-glucoside**	1 – 15	y = 17749 x + 65503	0.97	0.0303 ± 0.0110	1.0032 ± 0.0501
**Apigenin**	1 – 15	y = 98581x + 83264	0.99	0.2400 ± 0.0050	0.8002 ± 0.0168
		**Terpene**			
**Loganin**	1 – 15	y = 32797 x + 54032	0.99	0.0553 ± 0.0036	0.1846 ± 0.0118

[1] LOD: limit of detection.

[2] LOQ: limit of quantification.

Table S2. Effect sizes (η^2) of the type of extraction technique on the content of bioactive compounds in olive leaf.

Group of Compounds	η^2	95% CI[1] Lower	95% CI[1] Upper	Effect Size Interpretation
**Total compounds**	0.940	0.700	0.960	Very large
**Phenolic alcohols**	0.949	0.740	0.965	Very large
**Hydroxycinnamic acids**	0.872	0.429	0.914	Very large
**Flavonoids**	0.959	0.790	0.972	Very large
**Oleoside elenolic acid**	0.930	0.653	0.952	Very large
**Secoiridoids**	0.969	0.839	0.979	Very large
**Terpenes**	0.933	0.666	0.955	Very large

[1] CI: Confidence interval

Table S3. Quantification of compounds identified in dry weight (DW) of olive leaves.

COMPOUNDS	CE (mg compounds/g DW)	PLE (mg compounds/g DW)	SWE (mg compounds/g DW)	UAE (mg compounds/g DW)
**Phenolic acids**				
**Total phenolic acids**	**0.0666 ± 0.0043**	**0.0964 ± 0.0041**	**0.1054 ± 0.0105**	**0.1030 ± 0.0081**
Coumaric acid	0.0666 ± 0.0043	0.0964 ± 0.0041	0.1054 ± 0.0105	0.1030 ± 0.0081
**Phenolic alcohols**				
**Total phenolic alcohols**	**1.9394 ± 0.1676**	**3.4885 ± 0.6613**	**7.4201 ± 0.9848**	**2.6894 ± 0.1854**
Oxidized hydroxytyrosol isomer 1	0.1066 ± 0.0109	0.1032 ± 0.0041	0.0785 ± 0.0052	0.1156± 0.0057
Hydroxytyrosol glucoside	0.8419 ± 0.0787	1.1728 ± 0.2063	0.7396 ± 0.1468	1.1682 ± 0.0856
Hydroxytyrosol	0.8564 ± 0.1017	2.0630 ± 0.4362	6.0834 ± 1.0602	1.3406 ± 0.0917
Tyrosol	0.0481 ± 0.0081	0.1328 ± 0.0242	0.3401 ± 0.0556	0.0560 ± 0.0043
Oxidized hydroxytyrosol isomer 2	0.0863 ± 0.0149	0.0166 ± 0.0006	0.1785 ± 0.0359	0.0090 ± 0.0018
**Secoiridoids**				
**Total secoiridoids**	**15.4108 ± 1.6696**	**21.9891 ± 2.5521**	**2.7730 ± 0.2051**	**21.0888 ± 1.3494**
Hydroxyoleuropein	0.3449 ± 0.0517	0.5835 ± 0.1176	NQ	0.5120 ± 0.0507
Oleuropein isomer 1	4.4668 ± 0.3389	5.9792 ± 1.2035	ND	6.8827 ± 0.4303
Oleuropein diglucoside	0.1701 ± 0.0116	0.1789 ± 0.0266	0.0840 ± 0.0097	0.2433 ± 0.0134
Oleuropein isomer 2	5.4864 ± 0.5654	6.1971 ± 0.3143	1.8748 ± 0.2179	6.4982 ± 0.1314

Oleuropein isomer 3	3.8051 ± 0.5263	5.9904 ± 0.8440	ND	4.7779 ± 0.5715
Ligstroside isomer 1	0.2625 ± 0.0458	0.5840 ± 0.0981	ND	0.5821 ± 0.0703
Oleuropein isomer 4	0.1877 ± 0.0390	0.3599 ± 0.0751	0.2355 ± 0.0260	0.1513 ± 0.0217
Ligstroside isomer 2	0.3074 ± 0.0626	0.6713 ± 0.1368	0.2234 ± 0.0282	0.3975 ± 0.0384
Ligstroside isomer 3	0.0837 ± 0.0090	0.4340 ± 0.0828	ND	0.5187 ± 0.0614
Oleuropein isomer 5	0.2125 ± 0.0413	0.6437 ± 0.1301	0.1847 ± 0.0081	0.3521 ± 0.0202
Oleuropein isomer 6	0.0834 ± 0.0153	0.3671 ± 0.0771	0.1707 ± 0.0226	0.1731 ± 0.0401
**Flavonoids**				
**Total flavonoids**	**2.4441 ± 2.1344**	**3.8554 ± 0.3109**	**0.8073 ± 0.1244**	**4.9837 ± 0.6739**
Luteolin-7-glucoside isomer 1	0.3765 ± 0.0773	0.6950 ± 0.0827	0.3775 ± 0.0748	1.3305 ± 0.1571
Luteolin glucoside isomer 2	0.5385 ± 0.1027	0.8905 ± 0.1401	0.1342 ± 0.0250	1.1569 ± 0.1957
Luteolin glucoside isomer 3	0.0592 ± 0.0097	0.4482 ± 0.0671	ND	0.2335 ± 0.0473
Rutin	0.3325 ± 0.0471	0.1667 ± 0.1145	0.0763 ± 0.0140	0.4597 ± 0.0906
Luteolin glucoside isomer 4	0.7913 ± 0.1699	1.5876 ± 0.2974	0.2173 ± 0.0244	1.7197 ± 0.2220
Luteolin	0.0364 ± 0.0041	0.0674 ± 0.0147	0.0020 ± 0.0004	0.0835 ± 0.0115
Apigenin	NQ	NQ	NQ	NQ
**Phenylpropanoid/phenyletanol**				
**Total phenylpropanoid/ phenyletanol**	**0.1358 ± 0.0153**	**0.2077 ± 0.0382**	**-**	**0.3625 ± 0.0285**
Verbascoside	0.1358 ± 0.0153	0.2077 ± 0.0382	NQ	0.3625 ± 0.0285
**Oleoside and elenolic acid derivatives**				
**Total oleoside and elenolic acid derivatives**	**2.4825 ± 0.2858**	**2.9202 ± 0.5507**	**0.5311 ± 0.0291**	**2.9379 ± 0.2441**
Oleoside/secologanoside isomer 1	0.8102 ± 0.0958	0.8963 ± 0.1566	0.3800 ± 0.0323	0.9850 ± 0.0716
Glucopyranosyl acyclodihydroelenolic acid isomer 1	0.1002 ± 0.0089	0.0878 ± 0.0072	0.1102 ± 0.0033	0.0939 ± 0.0068

Oleoside/secologanoside isomer 2	1.1761 ± 0.1318	1.3162 ± 0.2716	0.0409 ± 0.0021	1.3323 ± 0.1207
Oleoside methyl ester / secologanoside methyl ester/ elenolic acid glucoside	0.3960 ± 0.0499	0.6199 ± 0.1174	NQ	0.5267 ± 0.0477
**Terpenes**				
**Total terpenes**	**0.5438 ± 0.0636**	**0.3956 ± 0.0784**	**0.2202 ± 0.0428**	**0.7373 ± 0.0601**
Loganic acid isomer 1	0.1113 ± 0.0089	0.0376 ± 0.0178	0.0240 ± 0.0051	0.1450 ± 0.0093
7-Epiloganin	0.0792 ± 0.0104	0.0771 ± 0.0140	0.0568 ± 0.0142	0.1065 ± 0.0104
Lamiol	0.2765 ± 0.0394	0.1570 ± 0.0312	0.125 6± 0.0217	0.3825 ± 0.0306
Loganic acid isomer 2	0.0585 ± 0.0094	0.1040 ± 0.0170	0.0138 ± 0.0024	0.0832 ± 0.0079
Maslinic acid	0.0182 ± 0.0018	0.0199 ± 0.0049	ND	0.0201 ± 0.0033

ND: non-detected, concentration below the LOD.

NQ: non-quantified, concentration below the LOQ.
